# Obesity indicators that best predict type 2 diabetes in an Indian population: insights from the Kerala Diabetes Prevention Program

**DOI:** 10.1017/jns.2020.8

**Published:** 2020-04-06

**Authors:** Nitin Kapoor, Mojtaba Lotfaliany, Thirunavukkarasu Sathish, K. R. Thankappan, Nihal Thomas, John Furler, Brian Oldenburg, Robyn J. Tapp

**Affiliations:** 1 Department of Endocrinology, Diabetes and Metabolism, Christian Medical College & Hospital, Vellore, Tamil Nadu, India; 2 Melbourne School of Population and Global Health, Faculty of Medicine, Dentistry and Health Science, The University of Melbourne, Melbourne, VIC, Australia; 3 Population Health Research Institute, McMaster University, Hamilton, Ontario, Canada; 4 Centre for Population Health Sciences, Lee Kong Chian School of Medicine, Nanyang Technological University, Singapore, Singapore; 5 Achutha Menon Centre for Health Science Studies, Sree Chitra Tirunal Institute for Medical Sciences and Technology, Trivandrum, Kerala, India; 6 Department of Public Health and Community Medicine, Central University, Kasaragod, Kerala, India; 7 Department of General Practice, Faculty of Medicine, Dentistry and Health Science, The University of Melbourne, Melbourne, VIC, Australia; 8 School of Biomedical Engineering and Imaging Sciences, King's College London, London, UK

**Keywords:** Obesity indicators, Type 2 diabetes mellitus, Visceral adiposity, Thin–fat phenotype, Normal-weight obesity, ROC, receiver operating characteristics, T2DM, type 2 diabetes mellitus, WC, waist circumference, WHR, waist:hip ratio, WHtR, waist:height ratio

## Abstract

Obesity indicators are known to predict the presence of type 2 diabetes mellitus (T2DM); however, evidence for which indicator best identifies undiagnosed T2DM in the Indian population is still very limited. In the present study we examined the utility of different obesity indicators to identify the presence of undiagnosed T2DM and determined their appropriate cut point for each obesity measure. Individuals were recruited from the large-scale population-based Kerala Diabetes Prevention Program. Oral glucose tolerance tests was performed to diagnose T2DM. Receiver operating characteristic (ROC) curve analyses were used to compare the association of different obesity indicators with T2DM and to determine the optimal cut points for identifying T2DM. A total of 357 new cases of T2DM and 1352 individuals without diabetes were identified. The mean age of the study participants was 46⋅4 (sd 7⋅4) years and 62 % were men. Waist circumference (WC), waist:hip ratio (WHR), waist:height ratio (WHtR), BMI, body fat percentage and fat per square of height were found to be significantly higher (*P* < 0⋅001) among those with diabetes compared with individuals without diabetes. In addition, ROC for WHR (0⋅67; 95 % 0⋅59, 0⋅75), WHtR (0⋅66; 95 % 0⋅57, 0⋅75) and WC (0⋅64; 95 % 0⋅55, 0⋅73) were shown to better identify patients with T2DM. The proposed cut points with an optimal sensitivity and specificity for WHR, WHtR and WC were 0⋅96, 0⋅56 and 86 cm for men and 0⋅88, 0⋅54 and 83 cm for women, respectively. The present study has shown that WHR, WHtR and WC are better than other anthropometric measures for detecting T2DM in the Indian population. Their utility in clinical practice may better stratify at-risk patients in this population than BMI, which is widely used at present.

India is currently home to 73 million people with diabetes and is projected to have the largest number in the world by 2045^(1)^. An even more alarming fact is that about 60 % of people with diabetes in India are unaware of their diagnosis^(1)^. A large proportion of these individuals with diabetes develop complications (including retinopathy, neuropathy and nephropathy), which can effectively be prevented by early diagnosis and treatment^(2–4)^. This suggests that appropriate identification and screening of high-risk individuals in a scalable and cost-effective approach at the primary-care level could have a significant public health impact. Among the several risk factors that help identify those with undiagnosed with diabetes, the presence of obesity is one of the most commonly used modifiable risk factors.

The rising prevalence of obesity has become a major public health concern and appropriate measurements to study its secular trend are essential. Precise epidemiological evaluation of obesity would depend on the type of obesity indicator used to measure it^(5)^. People in South Asian countries tend to develop type 2 diabetes mellitus (T2DM) at a much lower degree of obesity than those from other regions^(6,7)^. Recent evidence provided by the Global Burden of Disease study in 2017 estimated that less than 5 % of the Indian population is obese as defined by BMI (≥25 kg/m^2^), the most common indicator used to study obesity worldwide^(8)^. This paradox of having a large prevalence of patients with T2DM (up to 20 % in certain states) against a very low prevalence of obesity (as measured by BMI) may be partly explained by the inadequacy of BMI as an obesity indicator in this unique population. Further insights from recent literature suggest that South Asian populations may have a unique thin–fat phenotype, where they have more visceral obesity and high body fat content without much increase in BMI^(9)^. This unique body composition predisposes individuals to metabolic complications of obesity at a much lower BMI and may be better defined by another obesity indicator^(5,10)^.

Of the various obesity indicators that are available, BMI, which was first described by a Belgian mathematician in 1832, has been the conventional and the oldest indicator in use^(11)^. The other less commonly used obesity indicators include waist:hip ratio (WHR), waist circumference (WC), hip circumference and waist:height ratio (WHtR). More recently measuring body fat percentage, fat per square of height and visceral adipose tissue have been added to this armamentarium as they can now be measured using office-based equipment with adequate precision for clinical use. However, there is paucity of Indian literature to suggest which obesity indicator would best assess the presence of metabolic complications such as T2DM in this high-risk population.

Knowledge on the utility of obesity indicators that would best predict the presence of metabolic complications could increase our understanding of the discordance between a lower obesity prevalence and a large, rapidly increasing prevalence of diabetes^(12)^.

In this study we aimed to examine the utility of different obesity indicators to identify undiagnosed T2DM in an Indian population. We also aimed to determine the appropriate cut-off point for the most useful obesity indicator that may improve identification of high-risk individuals in this unique population.

## Material and methods

The Kerala Diabetes Prevention Program (K-DPP) is a cluster randomised clinical trial primarily designed to study the impact of a peer-led lifestyle intervention in reducing diabetes incidence among individuals at high risk for diabetes. The study was conducted in the Neyyattinkara taluk in Kerala's Trivandrum district in India and its study design is described in detail elsewhere^(13)^. This trial was approved by the Health Ministry Screening Committee of the Government of India, and ethics committees of the Sree Chitra Tirunal Institute for Medical Sciences and Technology (no. SCT/IEC-333/May 2011), Trivandrum, India; The University of Melbourne (no. 1441736) and Monash University (no. CF11/0457e2011000194) in Australia. The trial registration number at the Australia and New Zealand Clinical Trials Registry is ACTRN12611000262909. A written informed consent was taken from all participants prior to the initiation of the present study.

Normal healthy individuals from the community between the age of 30–60 years, recruited by a cluster random sampling method, were assessed for their sociodemographic characteristics (age, sex, occupation, education, marital status, household size and monthly household expenditure), lifestyle habits (diet, physical activity and substance use) and medical history using standardised questionnaires. Anthropometric measurements including height, weight, WC, hip circumference, WHR and WHtR were obtained using predefined standardised techniques^(14)^. Body composition was assessed using a TANITA body composition analyser (model SC330). This was used for the calculation of body fat percentage, fat per square of height and muscle mass per square of height. These measurements have a CV of about 5 % when compared with dual energy X-ray absorptiometry (DXA) scanning^(15)^. In addition, the oral glucose tolerance test was undertaken to diagnose the presence of T2DM. Those with a prior diagnosis of T2DM, myocardial infarction, stroke, arthritis, cancer, heart failure, epilepsy, dementia, or those currently using medications known to affect glucose metabolism (glucocorticoids, anti-psychotic drugs and anti-retroviral drugs) were excluded. Pregnant women were also excluded from participating in the study.

In this study we utilise the baseline screened participants of this trial for which in addition to clinical parameters they also had their body fat estimation and diabetes screening by methods outlined below^(13)^. Though the initial trial was conducted only among individuals with high Indian Diabetes Risk Score (IDRS) (>60), subsequently the same data were collected in individuals with low IDRS (<60), 3 years after the initial trial. Diabetes was defined by the criteria given by the American Diabetes Association following a 2-h 75 g oral glucose tolerance test. Individuals with a fasting plasma glucose value ≥126 mg/dl (≥7⋅0 mmol/l) and/or 2-h plasma glucose value of ≥200 mg/dl (≥11⋅1 mmol/l) were diagnosed to have diabetes. Other participants were grouped as people with no diabetes^(14)^.

The data collectors were given adequate training prior to the commencement of the study on data collection and a refresher training was given by the help of a training manual developed in line with the WHO STEPS (Stepwise approach to surveillance) training manual^(16)^.

### Statistical analysis

Data analysis was performed using STATA version 14.0 (StataCorp LP). Continuous variables with a normal distribution are presented as mean values and standard deviations. All continuous variables were compared across the groups (individuals with diabetes and those without diabetes) using independent *t* tests. The AUC were computed for each obesity indicator and T2DM, using receiver-operating characteristic (ROC) curves. ROC curves are a visual presentation of the relationship between sensitivity and specificity for a screening test and provide a simple tool for comparing the predictive power of different tools. ROC curve analyses and the respective AUC were used to compare the association of WC, hip circumference, WHR, WHtR, BMI, body fat percentage, fat per square of height and muscle mass per square of height with T2DM. The individual ROC were also compared independently with each other for the equality of the ROC area by the tested indicators. ROC curves were used to calculate the sensitivity, specificity and Youden's index, defined as ‘sensitivity + specificity – 1’ for the best obesity indicator as determined by the AUC. These determined the optimal values for predicting the presence of undiagnosed T2DM.

Considering the major statistical analysis used in this study as the comparison of AUC, we retrospectively calculated the power of the sample size by using the estimates of the parameters involved in the statistical tests^(17)^. Contemplating an α error of 0⋅05, with 357 T2DM cases and 1352 controls, the computed statistical power was 87⋅3 %. Therefore, the selected samples strongly support our analysis and conclusions.

## Results

The mean age of the study participants was 46⋅4 (sd 7⋅4) years and 62 % were men. A total of 357 new cases of T2DM and 1352 without diabetes were identified. Table 1 shows the study participant characteristics. Participants (men and women) with T2DM had significantly higher weight, WC, BMI, WHR and WHtR compared with participants without diabetes. Among indicators measured by bioelectrical impedance both fat and fat per height square were significantly higher in individuals with diabetes but there was no significant difference in muscle mass between those with and without diabetes, when assessed separately in men and women.

**Table 1.** Obesity indicators in participants with and without diabetes (Mean values and standard deviations; numbers of subjects)

	Participants without diabetes		Participants with diabetes		
	Mean	sd	Mean	sd	*P*
Subjects (*n*)	1352		357		
Height (cm)					
Men	165⋅6	8⋅8	165⋅6	6⋅9	0⋅681
Women	153⋅1	5⋅9	152⋅2	6⋅6	0⋅196
Weight (kg)					
Men	64⋅4	11⋅5	67⋅4	11⋅1	<0⋅001
Women	59⋅4	17⋅2	63⋅6	13⋅7	0⋅002
BMI (kg/m^2^)					
Men	23⋅3	3⋅6	24⋅5	3⋅3	<0⋅001
Women	25⋅3	4⋅3	27⋅3	5⋅1	0⋅001
Hip circumference (cm)					
Men	88⋅9	10⋅1	88⋅5	10⋅1	0⋅735
Women	96⋅6	11⋅1	97⋅3	12⋅3	0⋅644
Waist circumference (cm)					
Men	88⋅9	8⋅5	91⋅5	7⋅5	0⋅002
Women	88⋅1	11⋅1	95⋅0	12⋅4	0⋅002
Waist:hip ratio					
Men	1⋅01	0⋅09	1⋅04	0⋅07	0⋅001
Women	0⋅92	0⋅12	0⋅98	0⋅11	0⋅003
Waist:height ratio					
Men	0⋅53	0⋅05	0⋅55	0⋅04	<0⋅001
Women	0⋅58	0⋅07	0⋅62	0⋅08	0⋅001
Fat percentage					
Men	22⋅4	5⋅3	24⋅1	4⋅35	<0⋅001
Women	36⋅2	5⋅8	38⋅9	5⋅9	<0⋅001
Fat per square of height (kg/m^2^)					
Men	6⋅9	3⋅1	7⋅7	3⋅5	<0⋅001
Women	9⋅4	3⋅02	11⋅02	3⋅7	<0⋅001
Muscle mass per square of height (kg/m^2^)					
Men	16⋅3	2⋅1	16⋅6	2⋅2	<0⋅659
Women	15⋅0	1⋅2	15⋅4	1⋅5	<0⋅008

**Fig. 1.**
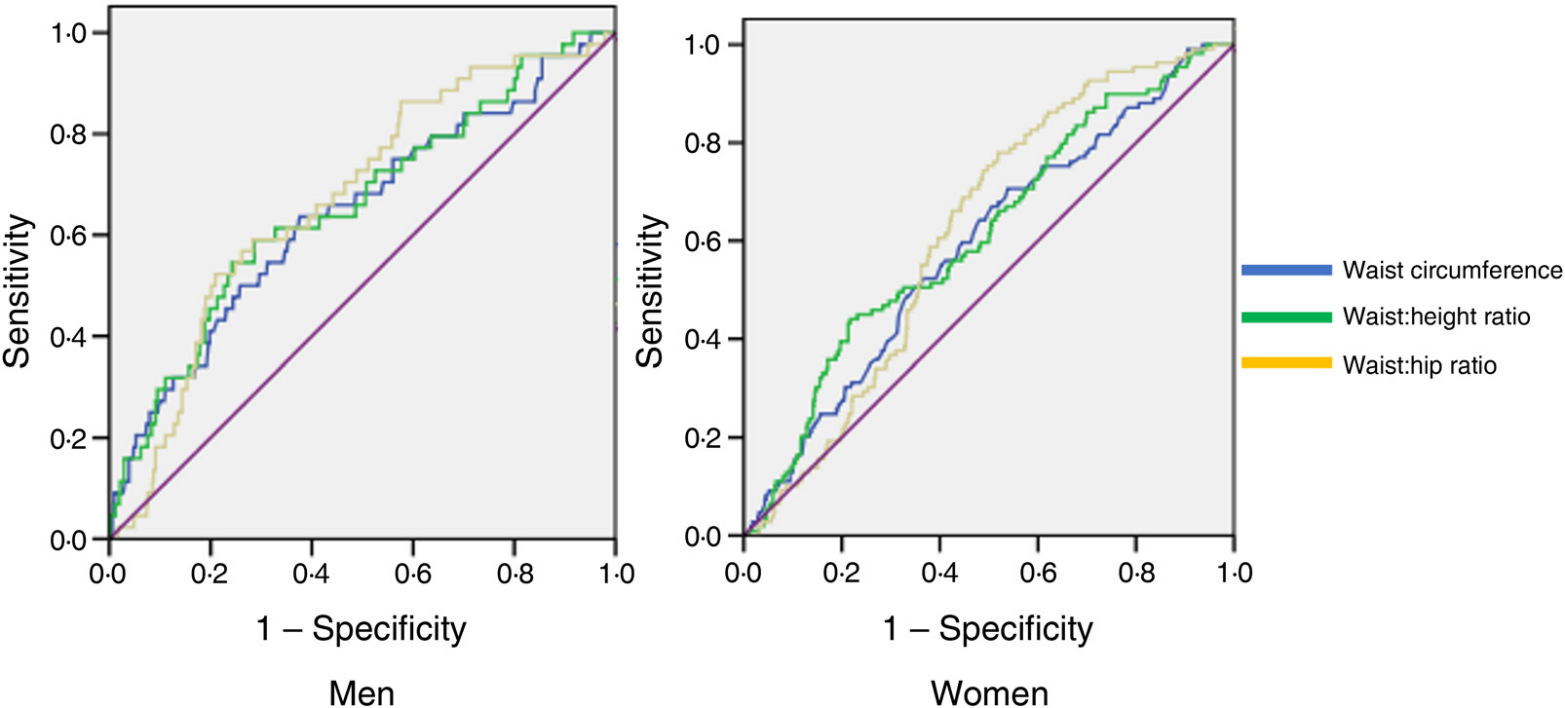
Receiver operating characteristic curves of anthropometric indicators in detecting type 2 diabetes mellitus in men and women.

Tables 2 and 3 indicate the AUC for different obesity indicators. These findings demonstrated that the association of WHR, WHtR and WC were higher than that for other indicators for T2DM and their detecting powers were similar in both men and women (Fig. 1). *P* values comparing the AUC for these parameters with other obesity indicators were also computed. In men, WHtR performed significantly better than weight, BMI, muscle mass per square of height, fat per square of height and fat percentage in identifying presence of diabetes (*P* < 0⋅001 for each measure). WC was more effective than BMI (*P* = 0⋅03), muscle mass per square of height (*P* = 0⋅03) and fat per square of height in men (*P* = 0⋅02). WHR and WC also performed better than weight in men (WHR & weight: *P* = 0⋅05; WC & weight: *P* < 0⋅001).

**Table 2.** Receiver operating characteristic (ROC) analysis for women

Indicator no.	Obesity indicator	AUC	se	95 % CI		Significance*
				Lower bound	Upper bound	
1	Waist:hip ratio	0⋅671	0⋅040	0⋅593	0⋅749	<0⋅001
2	Waist circumference	0⋅642	0⋅046	0⋅552	0⋅732	0⋅002
3	Waist:height ratio	0⋅657	0⋅045	0⋅570	0⋅745	0⋅001
4	Weight	0⋅569	0⋅048	0⋅474	0⋅663	0⋅13
5	BMI	0⋅593	0⋅044	0⋅507	0⋅678	0⋅04
6	Hip circumference	0⋅508	0⋅048	0⋅413	0⋅603	0⋅85
7	Body fat percentage	0⋅589	0⋅043	0⋅505	0⋅673	0⋅05
8	Fat per square of height	0⋅610	0⋅043	0⋅526	0⋅695	0⋅01
9	Muscle mass per square of height	0⋅580	0⋅045	0⋅492	0⋅668	0⋅078

* Significance to test the probability that the observed sample area under the ROC curve is >0⋅5 (rejecting the null hypothesis: area = 0⋅5 and if significant supported by the CI not crossing 0⋅5). Individual comparisons between ROC of different obesity indicators are included in the text.

**Table 3.** Receiver operating characteristic (ROC) analysis for men

Indicator no.	Obesity indicator	AUC	se	95 % CI		Significance*
				Lower bound	Upper bound	
1	Waist:hip ratio	0⋅650	0⋅020	0⋅611	0⋅690	<0⋅001
2	Waist circumference	0⋅605	0⋅024	0⋅559	0⋅652	<0⋅001
3	Waist:height ratio	0⋅588	0⋅023	0⋅542	0⋅634	<0⋅001
4	Weight	0⋅536	0⋅029	0⋅478	0⋅594	0⋅22
5	BMI	0⋅550	0⋅029	0⋅497	0⋅612	0⋅06
6	Hip circumference	0⋅485	0⋅029	0⋅427	0⋅543	0⋅61
7	Body fat percentage	0⋅561	0⋅030	0⋅501	0⋅620	0⋅04
8	Fat per square of height	0⋅554	0⋅030	0⋅495	0⋅613	0⋅07
9	Muscle mass per square of height	0⋅548	0⋅029	0⋅490	0⋅605	0⋅11

* Significance to test the probability that the observed sample area under the ROC curve is >0⋅5 (rejecting the null hypothesis: area = 0⋅5 and if significant supported by the CI not crossing 0⋅5). Individual comparisons between ROC of different obesity indicators are included in the text.

In women, WHtR and WC were shown to be better than weight, BMI and muscle mass per square of height in detecting the presence of undiagnosed T2DM. (*P* values for AUC comparison in women: WHtR & weight: *P* = 0⋅04; WHtR & BMI: *P* = 0⋅04; WHtR & muscle mass per square of height: *P* = 0⋅02; WHtR & fat percentage: *P* = 0⋅03; WC & weight: *P* = 0⋅03; WC & BMI: *P* = 0⋅03; WC & muscle mass per square of height: *P* = 0⋅05.) The comparisons of other obesity indicator ROC were not statistically significant.

ROC curve analyses for WC, WHR and WHtR were used to compare their discrimination in detecting undiagnosed T2DM (*P* < 0⋅001). We also assessed the optimal cut points for each of them for identifying diabetes in men and women. For women, 0⋅88 was the optimal WHR cut point in terms of Youden's index, and its sensitivity and specificity were 87 and 43 %, respectively. For men, the optimal cut-off point for WHR was 0⋅96, and its sensitivity and specificity were 83 and 40 %, respectively.

For women, the optimal WHtR cut point was 0⋅54 and for WC was 83 cm based on Youden's index. The sensitivity and specificity for WHtR were 82 and 82 % and for WC were 30 and 32 %. For men, the optimal cut point for WHtR was 0⋅56 and for WC was 86 cm. The sensitivity and specificity for WHtR were 82 and 75 % and for WC were 33 and 36 %.

## Discussion

This is the first study to comprehensively assess data on multiple obesity indicators (including those assessed by body composition analysis) and to determine their utility to predict the presence of undiagnosed T2DM in the phenotypically unique Indian population. Obesity indicators including WC, WHR, WHtR, BMI, body fat percentage and fat per square of height were shown to be higher in individuals with T2DM as compared with those without T2DM. Measures of central adiposity (WHR, WHtR and WC) each had a higher AUC than other obesity indicators for identifying individuals with T2DM. The proposed cut points with an optimal sensitivity and specificity for WHR were 0⋅96 in men and 0⋅88 in women, for WHtR 0⋅56 in men and 0⋅54 in women, and for WC 86 cm in men and 83 cm in women, in the Indian population.

This new knowledge on specific obesity indicators and their cut points in the Indian population provides vital information that is clinically relevant for general practitioners to screen for the metabolic risks of their patients quickly, easily and inexpensively. This information also provides further insights about the importance of fat distribution in the Indian population and its role in the pathogenesis of T2DM.

In the present study, we showed that central adiposity measures (WHtR, WC and WHR) were superior in identifying men and women with previously undiagnosed T2DM in the Indian population as compared with the other obesity indicators including BMI, which is currently widely used in clinical practice^(18)^. BMI probably did not perform superior in this study probably because of the unique phenotype of the south Asian population, who have a higher central adiposity even at lower BMI^(19)^. Wannamethee *et al*.^(20)^ showed that in a primary-care setting from the UK, WC and BMI had similar predictive power for identifying the presence of T2DM in older men, whereas WC was a superior predictor in European women. In another study in the Chinese population, WHR and WHtR were found to be better indicators in men, and WC and WHtR were better indicators in women, to identify individuals with undiagnosed T2DM^(21)^. In the Diabetes Prevention Program which included a more ethnically diverse study population, WC emerged as the most significant predictor of diabetes in both the lifestyle intervention and placebo group and this was irrespective of their sex^(22)^. Although the United States National Institute of Health clinical guidelines proposed WC for the evaluation of obesity as it does not require calculations, this has remained a matter of ongoing debate^(23,24)^. Moreover, the cut-off points for the use of WC in clinical practice also remain controversial in view of the ethnic variations and the differences in the proposed values^(25)^. There have been attempts to identify ethnicity-based cut points for WC; however, data with respect to those with Indian ethnicity is missing^(26)^.

A causal relationship between different obesity indicators and the occurrence of diabetes has also been shown by Mendelian randomisation studies^(27)^. Mendelian randomisation provides robust findings concerning the causal relationships of two variables based on the assumption of random distribution of alleles at conception. Genetic variants, thus detected, are used as unbiased proxy variables, and have shown a higher degree of obesity measured by different indicators with diabetes and other cardiometabolic disorders. These genetic variants are usually not related to confounding factors and cannot be altered by disease occurrence^(28)^.

Obesity indicators that were derived from body composition analysis were also measured in this study, though these parameters did not score as well as the clinical parameters. In the present study body composition was assessed using bioelectrical impedance, which does not distinguish between the metabolically active visceral adipose tissue from the more metabolically healthy subcutaneous fat and may explain the difference in findings^(29)^. This is especially relevant in the Indian population, in which a lower proportion of subcutaneous fat as compared with Caucasians has been described^(30)^. This in states of a positive energy balance leads to relatively increased fat deposition in the metabolically disadvantageous visceral adipose tissue. This phenomenon, called the fat overflow hypothesis, in particularly described in south Asian populations^(31)^. Similar findings have also been reported in a recently published study from Taiwan wherein they found the AUC to detect undiagnosed T2DM was higher for WC (0⋅745) and BMI (0⋅749) rather than total body fat percentage (0⋅687)^(32)^.

Visceral adipose tissue is not only implicated in the pathogenesis of diabetes, but also its reduction by acute restriction of dietary energy intake has now been shown to normalise β-cell function, hepatic glucose output and reverse diabetes in individuals with established T2DM^(4,33–36)^. This is mainly brought about by reduction in the pancreatic and hepatic fat content^(37)^. Presence of fat at ectopic sites is now considered as a key driver connecting adiposity with T2DM and other cardiometabolic disorders^(38,39)^.

Though definite cut-off points may help the practitioner to screen the high-risk individuals for metabolic complications, it is important to note that their influence on health risk is a continuum^(40)^. Our data indicated optimal discrimination for T2DM using obesity indicators like WHR, WHtR and WC. The thresholds proposed for these parameters are based on the best possible balance between sensitivity and specificity. Our cut points identify risk factors with a sensitivity greater than 80 % and specificity greater than 40 %, whereas for BMI the specificity drops to only 15 % at similar sensitivity. It can offer an alert about the practical boundary for initiating intervention to prevent and screen for the risk of T2DM using obesity indicators in a primary-care Indian setting. The cut-offs derived in present study are comparable with previously published literature (WC men: 89 cm; women: 83 cm; WHtR men: 0⋅52; women: 0⋅51; WHR men: 0⋅89; women: 0⋅81)^(41)^.

We acknowledge that the AUC obtained in the present study are not >0⋅8, as may be desirable for an ideal screening tool; however, the results of this study do emphasise the importance of changing the conventional practice of using only BMI at the primary-care level by using other more useful obesity indicators, especially in this population. There are several plausible reasons that explain a relatively lower yet significant AUC for the obesity indicators in this study, despite the well-known fact that obesity is associated with T2DM. One of the important reasons that is unique to this population is the inability of any of these measures to differentiate the presence of visceral adipose tissue against subcutaneous adipose tissue^(5)^. A disproportionately lower subcutaneous adipose tissue has been described in the south Asian population^(31)^. Other risk factors when used in conjunction with appropriate obesity indicators have better AUC^(42,43)^.

The results from the present study would be useful for health professionals to identify the high-risk individuals belonging to Indian ethnicity, for efficient screening of diabetes at the primary-care level. This information would also assist the health authorities and policy makers to develop ethnicity-specific screening guidelines based on obesity indicators that would best identify high-risk individuals in the community for appropriate intervention.

This is a unique study to assess for the first time data on multiple obesity indicators to predict the presence of diabetes in the Indian population. The results of the present study are based on the baseline findings of the Kerala Diabetes Prevention Program (K-DPP) dataset, which is an ideal cohort to study the relevance of obesity indicators in the Indian population, as it followed standardised anthropometric measurements from individuals who were taken from the community and have had appropriate screening tests for the diagnosis of T2DM. Data on obesity indicators measured through body composition analysis were also distinctive in this study, as limited literature is available on these parameters from the Indian population.

The main limitation in this study is that it is a cross-sectional study, and we cannot draw conclusions about cause-and-effect relationships between obesity indicators and T2DM. Our participants also came from the southern region of India and therefore may not be representative of the Indian population. Also, a part of the population with low Indian Diabetes Risk Scores had their measurements 3 years after the initial evaluation, which may have introduced a bias in terms of progression of age-related co-morbidities. We acknowledge that the study population consists of a larger proportion of men and the cut-offs obtained for men are probably more robust.

### Conclusion

The present study has shown that WHR, WHtR and WC are better than other anthropometric measures for detecting T2DM in the Indian population. Their utility in clinical practice may better stratify at-risk patients in this population than BMI, which is widely used at present.
